# Regulation of fruit and seed response to heat and drought by sugars as nutrients and signals

**DOI:** 10.3389/fpls.2013.00282

**Published:** 2013-08-01

**Authors:** Yong-Hua Liu, Christina E. Offler, Yong-Ling Ruan

**Affiliations:** ^1^Department of Biology, School of Environmental and Life Sciences, The University of NewcastleNewcastle, NSW, Australia; ^2^Institute of Vegetables, Zhejiang Academy of Agricultural SciencesHangzhou, China

**Keywords:** sugar metabolism and signaling, fruit and seed set, heat and drought, cell division, hormones, programmed cell death, reactive oxygen species, non-enzymic antioxidant

## Abstract

A large body of evidence shows that sugars function both as nutrients and signals to regulate fruit and seed set under normal and stress conditions including heat and drought. Inadequate sucrose import to, and its degradation within, reproductive organs cause fruit and seed abortion under heat and drought. As nutrients, sucrose-derived hexoses provide carbon skeletons and energy for growth and development of fruits and seeds. Sugar metabolism can also alleviate the impact of stress on fruit and seed through facilitating biosynthesis of heat shock proteins (Hsps) and non-enzymic antioxidants (e.g., glutathione, ascorbic acid), which collectively maintain the integrity of membranes and prevent programmed cell death (PCD) through protecting proteins and scavenging reactive oxygen species (ROS). In parallel, sugars (sucrose, glucose, and fructose), also exert signaling roles through cross-talk with hormone and ROS signaling pathways and by mediating cell division and PCD. At the same time, emerging data indicate that sugar-derived signaling systems, including trehalose-6 phosphate (T6P), sucrose non-fermenting related kinase-1 (SnRK), and the target of rapamycin (TOR) kinase complex also play important roles in regulating plant development through modulating nutrient and energy signaling and metabolic processes, especially under abiotic stresses where sugar availability is low. This review aims to evaluate recent progress of research on abiotic stress responses of reproductive organs focusing on roles of sugar metabolism and signaling and addressing the possible biochemical and molecular mechanism by which sugars regulate fruit and seed set under heat and drought.

## Introduction

Food security is becoming a more and more important and urgent issue with increasing demand for enhancement of crop yield. It is estimated that more than 1 billion people are currently undernourished worldwide (Garrity et al., 2010; Godfray et al., 2010) and the world population is predicted to reach more than 9 billion by 2050 (Fedoroff et al., 2010). To meet this population growth crop yield must be doubled by the middle of this century (Beddington, 2010). Unfortunately, the arable land area for food production is rapidly decreasing because of urbanization, salinization, desertification, and competition from biofuel production (Döös, 2002; Solomon, 2010). This means we have to significantly increase crop yield per unit land area to meet the increasing food demand.

Increasing crop yield is becoming more challenging with global warming. It has been predicted that there will be 1.5–5.8°C increase in average annual temperatures by 2100 (Zinn et al., 2010) and approximately 2.5–16% yield loss for every 1°C increase in temperature above optima (Battisti and Naylor, 2009). Furthermore, in field conditions, drought often occurs simultaneously with heat and the combination of drought and heat can lead to a synergistic detrimental effect on crop productivity (Rampino et al., 2012).

The development of reproductive organs (mainly fruits and seeds and their precursors, ovaries and ovules) plays a dominant role in global crop production. In the top fifteen major crops worldwide, there are 10 crops that are consumed as fruit and seed crops (Ross-Ibarra et al., 2007). Generally, about 75% of the total worldwide crop yield comes from fruit and seed crops (Ruan et al., 2010). Fruit and seed yield is mainly determined by fruit and seed number and their size. Fruit and seed abortion is a major limiting factor for achieving crop yield potential (Boyer and McLaughlin, 2007; Patrick and Stoddard, 2010; Ruan et al., 2012). In the plant life cycle, the early stage of fruit and seed development at the set phase is one of the most sensitive periods to abiotic stresses such as heat, drought, and cold (Barnabás et al., 2008; Hedhly et al., 2008; Thakur et al., 2010). Abiotic stresses during the early reproductive stage often cause abnormal development of reproductive organs that results in failure of fertilization or abortion of fruits and seeds (Thakur et al., 2010), thereby dramatically decreasing crop yield (Setter et al., 2011; Kakumanu et al., 2012). For example, drought at the flowering stage caused severe kernel abortion in maize (McLaughlin and Boyer, 2004a). In tomato, flower abortion rate can reach up to 80% under heat stress (Ruan et al., 2010). Similarly, seed set of *Brassica napus* was reduced by 88% by heat stress (Young et al., 2004). Thus, increasing fruit and seed set under various abiotic stresses is a viable option for sustaining crop yield in the face of climate change. Despite the importance and sensitivity of the fruit and seed set processes, most research effort in fruit and seed biology to date has been dedicated to the late stage of growth and maturation of fruit and seed (Wang et al., 2009), and little is known about the physiological and molecular mechanisms regulating fruit and seed set under abiotic stresses (Ruan et al., 2012).

## Sugar metabolism in fruit and seed development

Fruit and seed development depends on import of sugars in the form of sucrose transported through phloem from source leaves in most species (Egli, 2010; Foulkes et al., 2011) since fruit photosynthesis is negligible in terms of its contribution of assimilates to fruit development (Blanke and Lenz, 1989). This conclusion is supported by a recent molecular study where the specific suppression of glutamate 1-semialdehyde aminotransferase (GSA) in fruits, a key enzyme in chlorophyll biosynthesis, had no effect on fruit growth and ripening (Lytovchenko et al., 2011). However, fruit photosynthesis plays vital roles in early seed development since seed set was seriously compromised in the transgenic plants, indicating early seed development is more sensitive to reduction of carbon supply than fruit. Before participating in various physiological and metabolic processes, phloem-unloaded sucrose must be degraded into hexoses (glucose and fructose) or their derivates by sucrose synthase (Sus, EC 2.4.1.13) or invertase (INV, EC 3.2.1.26) (Sturm, 1999). Sus is a glycosyl transferase, which reversibly converts sucrose in the presence of UDP into UDP-glucose and fructose, whereas INV irreversibly hydrolyses sucrose into glucose and fructose. Based on their subcellular location, INVs can be classified into three subgroups: cell wall invertase (CWIN), vacuolar invertase (VIN), and cytoplasmic invertase (CIN) (Sturm, 1999).

Sugar metabolism provides not only energy to power numerous cellular processes, but also substrates for biosynthesis of biopolymers such as starch, cellulose, callose, and protein. At the same time, sucrose metabolism in sink organs can help to establish sink strength by lowering sucrose concentration in recipient sink cells thereby facilitating sucrose import from source to sink (Ho, 1988). Furthermore, hexose produced by INV and Sus-mediated sucrose degradation can act as a signaling molecule to regulate plant development (Ruan, 2012). It has been suggested that INV and Sus may play particularly important roles in bulky organs such as fruits and seeds in many crop species as compared to that in their wild progenitors (Xu et al., 2012). For example, the loss of a functional CWIN in maize kernels resulted in a miniature seed phenotype (Miller and Chourey, 1992). Similarly, specific overexpression of CWIN in rice by its native promoter increased grain yield (Wang et al., 2008). In contrast, silencing CWIN expression in tomato resulted in increased fruit abortion, and reduced fruit size and seed number per plant (Zanor et al., 2009).

## Central roles of sugar metabolism in fruit and seed set under heat and drought

Compared to vegetative organs, young reproductive organs are less competitive for nutrient acquisition, which may result from their distal location from source leaves, lower transport conductivities through plasmodesmata and differentiating phloem and their low INV activities (Ruan et al., 2012). Thus, even under optimal conditions, sugar availability could become one limiting factor for fruit and seed set (Ghiglione et al., 2008).

Under heat and drought, sugar limitation is a well-known factor leading to fruit and seed abortion (Boyer and McLaughlin, 2007; Barnabás et al., 2008). For example, as a result of heat stress, the growth rate of pollen tubes through the style of cotton is limited by an inadequate supply of sucrose and hexose in the pistil (Snider et al., 2011). In maize, drought resulted in severe ovary abortion, while feeding sucrose to the stems of water-stressed plants partially prevented the abortion and restored kernel number (Zinselmeier et al., 1999; McLaughlin and Boyer, 2004a) demonstrating that low availability of sucrose or hexose is a casual factor of ovary abortion. Further analyses revealed that ovary abortion was linked more closely to the availability of glucose than sucrose in the ovary (McLaughlin and Boyer, 2004a), since sucrose concentrations were completely restored after sucrose feeding but glucose concentrations were only partially restored (Zinselmeier et al., 1999). Therefore, inefficient conversion of sucrose to glucose and fructose is a key limiting step for ovary development under drought. In fact, McLaughlin and Boyer (2004b) showed that sucrose feeding only partially restored the activity of invertase which was decreased under drought. Consistently, silencing of CWIN gene (*Lin5*) in tomato increased fruit abortion under drought (Zanor et al., 2009). On the other hand, elevation in CWIN activity through silencing expression of the CWIN inhibitor in tomato delayed leaf senescence and enhanced fruit and seed development (Jin et al., 2009). It will be important to determine if these CWIN-elevated transgenic plants exhibit increased tolerance to abiotic stress.

## Possible mechanisms underlying regulation of fruit and seed set by sugars as nutrients under heat and drought

Sucrose metabolism not only provides energy and carbon skeletons for sink development, but also regulates their response to abiotic stresses by providing hexoses as essential metabolites and signaling molecules (Ruan et al., 2012).

### Regulation of carbon partitioning

Mild heat stress before silking enhanced the total biomass of maize plants, but with reduced grain yield (Suwa et al., 2010) which indicates that carbon partitioning favors vegetative organs over reproductive tissues when under stress. The difference in response to abiotic stresses between reproductive and vegetative organs may be related to different responses of sucrose degrading enzymes to stress conditions. For example, drought induced the expression of VIN (*Ivr2*) in vegetative tissues of maize, but reduced the expression of *Ivr2* in reproductive organs (Kim et al., 2000). Similar result was also observed in soybean in which drought decreased the activity of soluble invertase in pods, but not that in leaves (Liu et al., 2004). By using RNA-Seq analysis, Kakumanu et al. (2012) found that there are more genes related to carbohydrate metabolism responsive to drought in the maize ovary than in young leaves. Among them, one gene encoding sucrose synthase showed decreased expression in the ovary, but not in leaf meristems, under drought. This may decrease the sink strength of these reproductive organs leading to sucrose partitioning in favor of vegetative tissues (Sturm and Tang, 1999; Andersen et al., 2002). Consistent with this postulation, a heat tolerant tomato genotype exhibited higher CWIN and VIN activities in the flower and young fruit and consequently a higher rate of sucrose import into young fruit than a heat sensitive genotype, which collectively contributed to higher fruit set under heat stress (Li et al., 2012). Thus, a high ability of sucrose partitioning to, and its degradation within, reproductive organs could be vital for fruit and seed set under heat and drought.

### Sucrose metabolism contributes to antioxidant protection

Heat and drought often lead to excess accumulation of reactive oxygen species (ROS), including singlet oxygen, superoxide (O_2_^−^), hydrogen peroxide (H_2_O_2_), and the hydroxyl radical (OH^−^), which may result in oxidative damage of DNA, proteins, and lipids (Oktyabrsky and Smirnova, 2007), and finally programmed cell death (PCD) and fruit and seed abortion (Laloi et al., 2004; Vacca et al., 2004; Foyer and Noctor, 2005). It has been proposed that sugar limitation caused by various stresses might be an important basis for ROS accumulation (Couée et al., 2006; Bolouri-Moghaddam et al., 2010). Glucose metabolism may play positive roles in preventing PCD by scavenging ROS. As the primary carbon and energy source in plants, glucose can feed the oxidative pentose phosphate pathway and produce reducing power for biosynthesis of non-enzymic antioxidants such as glutathione (GSH), ascorbic acid (Asc), phenolic compounds, and flavonoids (Bolouri-Moghaddam et al., 2010), which can efficiently scavenge ROS. Glucose-6-phosphate dehydrogenase (G6PDH) is the rate-limiting enzyme in the oxidative pentose phosphate pathway. A study on soybean showed that G6PDH plays a central role in maintaining the ROS homeostasis under drought stress through increasing the activities of glutathione reductase (GR), dehydroascorbate reductase (DHAR), and monodehydroascorbate reductase (MDHAR) and the content of GSH and Asc (Liu et al., 2013). In addition, oxidative stresses caused by high light and herbicide, paraquat, can activate antioxidant response in Arabidopsis plants (Sunkar et al., 2006). However, exogenous addition of sucrose to Arabidopsis reduced the expression of SOD genes (*CSD1* and *CSD2*) under high light and paraquat, implying sucrose might prevent oxidant stress to some extent (Dugas and Bartel, 2008). Furthermore, some soluble sugars, such as fructan, might act as ROS scavengers themselves when they exist at high concentration (Van den Ende and Valluru, 2009). Further evidence on sucrose metabolism alleviating oxidative stress comes from transgenic potato plants overexpressing yeast INV that displayed lower malondialdehyde (MDA) content under cold stress (Sinkevich et al., 2010). This indicates INV may improve cold tolerance through enhancing the antioxidant capability of potato plants (Sinkevich et al., 2010). Another example is that oxidative stress such as that caused by application of H_2_O_2_ often increases the expression of ascorbate peroxidases (APXs), enzymes playing important roles in maintaining the plant's antioxidant system. Overexpressing CIN in Arabidopsis protoplasts, however, alleviates the elevation of APX expression upon H_2_O_2_ application, which implies that CIN may ameliorate oxidative stress directly (Xiang et al., 2011). Although the majority of these studies were conducted in vegetative tissues, it can be envisaged that sugar metabolism may play positive roles in fruit and seed set through maintaining ROS homeostasis under heat and drought.

### Sucrose metabolism and heat shock proteins (Hsps)

Sugar metabolism may also ameliorate effects of abiotic stresses by fueling biosynthesis of heat shock proteins (Hsps). Under heat and drought, Hsps are often induced to prevent or attenuate stress-induced PCD though preserving membrane integrity and protein function. Severe heat stress could lead to the hyperfluidization and disruption of membranes (Horváth et al., 1998; Sangwan et al., 2002), inactivation of proteins by unfolding, misfolding, and aggregation (Sharma et al., 2010) and the accumulation of ROS (Dat et al., 1998; Volkov et al., 2006). These detrimental effects collectively damage photosynthesis and retard plant growth and development and may even cause death of sensitive tissues (Mittler and Blumwald, 2010). During evolution, plants have developed a system of heat-inducible cellular and molecular defences, i.e., the heat stress response (HSR). During HSR, hundreds of specific genes become up-regulated (Mittler et al., 2012). Among them, accumulation of Hsps is a major feature of HSR. Many of the Hsps are chaperones, including Hsp100s, Hsp90s, Hsp70s, Hsp60s, and small Hsps (sHsps) (Finka et al., 2011). Chaperones can prevent protein aggregation or restore misfolded and aggregated proteins to correctly-folded polypeptides. These polypetides can recover their active conformation for normal function after stress exposure (Sharma et al., 2010). Thus, Hsps play an important role in plant heat tolerance. Indeed, constitutive overexpression of Hsp17.7 in carrot (Malik et al., 1999) or Hsp101 in Arabidopsis (Queitsch et al., 2000) increased their heat tolerance.

Studies in animal systems showed that Hsp chaperones (e.g., Hsp70 and sHsps) can block PCD (Beere, 2004; Weiss et al., 2007). In Arabidopsis, *CNGC2* encodes a component of cyclic nucleotide gated Ca^2+^ channels which are a kind of thermosensor in land plants. *CNGC2* mutants accumulated Hsps at lower heat-priming temperatures and consequently have higher heat tolerance and less PCD than wild-type plants (Finka et al., 2012). Surprisingly, *CNGC2* mutants showed growth retardation under control temperature, but without other detectable cellular, morphological, or developmental defects. It has been suggested that this may result from insufficient supply of photoassimilates since maintaining of HSR comes at a high energetic and metabolic cost (Finka et al., 2012; Mittler et al., 2012). Support for this hypothesis comes from a recent finding that heat-stress induced expression of two HSP genes, *LeHSP17.4-CII* and *LeHSP17.6-CII*, correlates with enhanced transcript levels and activities of VIN in 5-d tomato fruit (Li et al., 2012). However, evidence is still lacking as to whether, and to what extent, induction of Hsps is dependent on glucose metabolism and signaling that is coupled with INV activity.

## Possible mechanisms underlying regulation of fruit and seed set by sugars as signals under heat and drought

Sucrose metabolism also plays signaling roles in many developmental processes (Ruan, 2012), where sucrose and its degrading products, glucose and fructose, can act as signaling molecules to regulate gene expression (Moore et al., 2003; Wind et al., 2010; Cho and Yoo, 2011). However, it is difficult to distinguish the specific signaling role of sucrose from hexoses since sucrose can be quickly degraded into glucose and fructose. Thus, although the role of sucrose as a signaling molecule in plants was proposed several decades ago, it has been experimentally established and accepted only recently based on emerging evidence (Tognetti et al., 2013). For example, application of exogenous sucrose to the aerial part of Arabidopsis plants facilitate the initiation of lateral roots, but equal molar concentrations of glucose or fructose promote lateral root formation to a much lesser extent (Macgregor et al., 2008). Most recent studies on strawberry (*Fragaria* × *ananassa*) show that sucrose is the key signaling molecule in fruit ripening (Jia et al., 2013). Exogenous sucrose increased ABA content in fruit and accelerated fruit ripening which can be mimicked by turanose, a sucrose non-metabolizable analog, implying the possible signaling role of sucrose in fruit ripening. Their further studies revealed that silencing of the sucrose transporter (*FaSUT1*) by RNAi technique decreased sucrose level and blocked fruit ripening, whereas overexpression of *FaSUT1* increased sucrose level and accelerated fruit ripening (Jia et al., 2013). However, no sucrose-specific sensor has been identified thus far (Wind et al., 2010), and little is known about the nature of sucrose signaling and the regulatory pathways modulated by sucrose.

Recently, it has been suggested that hexoses derived from sucrose cleavage by Sus and INV play important roles in plant development through signaling pathways (Koch, 2004; Ruan, 2012). However, up to now, research on sugar signaling has mainly focused on glucose (Smeekens et al., 2010; Cho and Yoo, 2011). Although fructose is also an abundant hexose produced from both INV- and Sus-catalysed sucrose degradation, little is known about its potential signaling role. Only recently, a systematic study was done in Arabidopsis which revealed that fructose signaling plays an important role in seedling development and fructose-1, 6-biphosphatase was identified as the fructose sensor in the signaling pathway (Cho and Yoo, 2011).

In addition to the above mentioned sugar signaling (i.e., sucrose, glucose, and fructose), the importance of some sugar-derived signaling systems in plant development is becoming increasingly prominent. These systems include the trehalose 6-phosphate (T6P) signal, and the target of rapamycin (TOR) kinase system, the SNF1-related protein kinase (SnRK) and bZIP transcription factor network. Here, we will first focus on the recent progress about the regulatory role of sugar (mainly glucose) signaling in fruit and seed development under abiotic stresses followed by discussion of the possible roles of T6P, TOR, SnRK1, and bZIP in plant development under abiotic stress.

### Crosstalk between sugar- and hormone-signaling pathways: a general phenomenon

There is compelling evidence on crosstalk between sugar- and hormone-signaling pathways (LeClere et al., 2010; Ruan, 2012). For example, increased petal and sepal number in tomato in response to inhibition of CWIN gene (*Lin5*) expression is linked to decreased ABA, JA and GA levels (Zanor et al., 2009). Most studies focus on the positive interaction between sugars and auxins, an important hormone in plant development. The Arabidopsis hexokinase (HXK) mutant *gin2* (*glucose insensitive 2*) is less sensitive to exogenous auxin indicating that the glucose-signaling pathway interacts with, or may act downstream of, the auxin signaling pathways through the HXK-dependent pathway (Moore et al., 2003). On the other hand, a recent study on Arabidopsis provides direct evidence that auxin biosynthesis is tightly dependent on endogenous glucose level (Sairanen et al., 2012). Consistently, exogenous glucose up-regulated the expressions of auxin biosynthetic genes (*YUCCA*) and IAA transporter genes (*PINs*) in roots of young Arabidopsis seedlings, and an auxin receptor mutant (*tir1*) and response mutants (*axr2, axr3* and *slr1*) showed a defect in glucose-induced root elongation and lateral root production (Mishra et al., 2009). Decrease of hexose level in basal regions of maize kernels by deficiency of CWIN activity results in reduced IAA levels in miniature kernels that are related to suppressed expression of the IAA biosynthesis gene (*ZmYUC*) through a HXK-dependent pathway (LeClere et al., 2010). There is also a positive relationship between sugar and cytokinin signaling. For example, delayed leaf senescence by cytokinin depends on expression of CWIN in tobacco leaves (Lara et al., 2004), which implies a positive relationship between sugar and cytokinin signaling.

A negative interaction exists between glucose and ethylene through ETHYLENE-INSENSITIVE3 (EIN3), a key transcriptional regulator in ethylene signaling (Chao et al., 1997). Glucose can suppress the activity of EINs by enhancing their degradation through ubiquitination. Importantly, the non-signaling glucose analog, 3-*O*-methyl-glucose (3-OMG) which can not be phosphorylated by HXK in plants, did not suppress the activity of EIN3. This finding indicates that glucose acted as a signal molecule to induce the degradation of EIN3 in a HXK dependent manner (Yanagisawa et al., 2003). Indirect evidence also showed a negative interaction between sugars (hexoses) and ABA. For example, increased CWIN activity by silencing expression of CWIN inhibitor (*LeINVINH1*) delayed ABA-induced leaf senescence in tomato, which suggests ABA-mediated senescence is dependent on decreased CWIN activity, hence possibly a lower glucose or fructose level in the apoplasm (Jin et al., 2009). Interestingly, a study on rice anthers showed that ABA accumulation, prior to decreased CWIN and monosaccharide transporter expression, is the initial event responsible for pollen sterility under cold treatment (Oliver et al., 2007). Similarly, an increase in ABA level in maize ovaries occurred immediately after drought was imposed which is followed by a decrease in expression of soluble invertase (*Ivr2*) (Andersen et al., 2002). However, Pinheiro et al. (2011) found that a change in carbohydrate metabolism rather than in ABA level is the initial response of *Lupinus albus* to slowly imposed drought. This contradictory conclusion may be resulted from variability in type and strength of stress imposed.

Reciprocally, hormones can also affect sugar metabolism. For example, exogenous supplement of NAA to tobacco cells stimulated the activity of CWIN (Weil and Rausch, 1990). The induction of CWIN by cytokinins was observed in suspension cell cultures of *Chenopodium rubrum* (Ehness and Roitsch, 1997), and the result is further supported by the induction of CWIN activity by an endogenous increase of cytokinin in tobacco (Lara et al., 2004). Exogenous ABA decreased the CWIN activity in tomato leaves mainly through increasing the expression of CWIN inhibitor (*INVINH1*) (Jin et al., 2009). Overall, there appears to be synergistic relationships between CWIN activity and growth-related hormones (auxin, cytokinin), but antagonistic relationships between CWIN and senescence hormones (ABA and ethylene).

### Sugar signaling and cell division in fruits and seeds under heat and drought stress

Early evidence about sugar signaling in reproductive organs comes from the observation that hexoses stimulate cell division, while sucrose promotes cell endoreduplication and starch accumulation in *Vicia faba* cotyledons (Weber et al., 1996). These authors further proposed that CWIN in the seed coat of *Vicia faba* can affect the developmental processes of seeds through regulating sugar signaling in the embryo. In maize, mutation of a CWIN gene (*INCW2*) resulted in a miniature seed phenotype by blocking endosperm cell division (Vilhar et al., 2002). Further, Baldet et al. (2006) suggested that the possible mechanism for carbohydrate control of tomato fruit size is through the regulation of cell proliferation. The effect of sugar signaling on cell division in seed and fruit may be realized through their regulation of cyclins (e.g., cyclin D—Dewitte and Murray, 2003; Weber et al., 2005). It has been suggested that decreased glucose level under stress conditions could repress cell division and consequently lead to fruit and seed abortion (Ruan et al., 2012). A recent study showed that drought inhibited the expression of two main invertase genes, *Incw1* and *Incw2*, in the maize ovary and decreased the hexose level (Kakumanu et al., 2012). It has been suggested that this reduced hexose level arrested cell division through decreasing the expression of cyclins and increasing the expression of the cyclin-dependent kinase inhibitor (CDKI). Consistently, in the more resistant leaf meristem, drought increased invertase expression, which maintained the hexose level and cell division activity.

**Figure 1:**
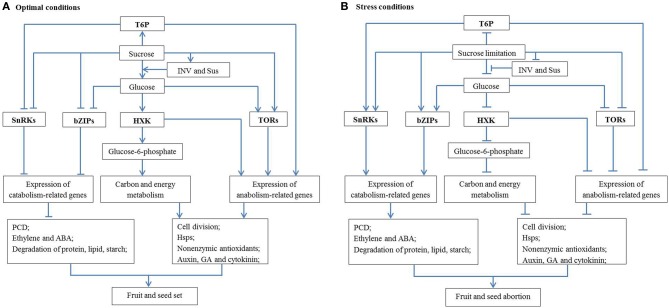
**A schematic network illustrating the mechanisms by which sugar metabolism and signaling regulate fruit and seed set under abiotic stresses. (A)** Under optimal conditions with high sucrose availability, INV and Sus activities generate sufficient amount of hexoses (glucose) to activate cell division; to facilitate the production of non-enzymatic antioxidants and to inhibit PCD. On the other hand, T6P promotes biosynthetic process and represses the activity of a growth inhibitor, SnRKs. These features interact with hormonal signaling pathways to allow seed and fruit set and subsequent growth to proceed. **(B)** The reverse applies under stress conditions where sugars are limited, and fruits and seeds abort. Arrow and T-type line represent the positive and negative effect, respectively. See text for more details. HSP, heat shock protein; HXK, hexokinase; INV, invertase; PCD, programmed cell death; Sus, sucrose synthase; T6P, trehalose-6-phosphate; TOR, target of rapamycin; SnRK, sucrose non-fermenting-1 (SNF1)-related protein kinases; bZIP, basic leucine-zipper proteins.

Sugar signaling regulation of cell division might be realized through crosstalk with hormone signaling. It has been proposed that normal fruit and seed development relies on induction of auxin, GA and cytokinin responses and attenuation of ethylene and ABA responses (Dorcey et al., 2009; Ji et al., 2011; Ruan et al., 2012). Auxin, GA and cytokinin can facilitate fruit and seed set and development by enhancing cell division and cell expansion (Gillaspy et al., 1993), whereas ethylene and ABA are senescence and stress hormones which can hamper fruit and seed development (Davies, 2010). It can be postulated that, therefore, under optimal conditions, glucose signals can promote cell division, and consequently fruit and seed set through enhancing growth-related hormone signaling (auxin, GA and cytokinin, Figure 1A). Under stress conditions, however, decreased glucose levels resulting from reduced sucrose import into, and INV activity within, ovaries and seeds may lead to activation of senescence hormone signaling (ethylene and ABA) and arrest cell division, and finally fruit and seed abortion (Figure 1B).

### Crosstalk between sugar- and ROS-signaling pathways

As detailed above, excess ROS will damage DNA, proteins and lipids. However, ROS also play a signaling role at lower concentrations, which is essential for many developmental or metabolic processes such as PCD, cell wall biosynthesis, and stress responses (Kovtun et al., 2000; Mittler, 2002; Andriunas et al., 2012). An important mechanism of ROS signaling is to regulate the interaction between proteins through altering the redox state of amino acids such as cysteine (Cys). ROS can also play their signaling role through calcium signaling and affecting transcription factors (Munné-Bosch et al., 2013). Among all the ROS species, H_2_O_2_ is the most common signaling molecule since it is relatively stable and water-soluble (Bienert et al., 2007; Van Breusegem et al., 2008). Under stress conditions, maintaining ROS at an optimal level can facilitate their beneficial signaling function without causing oxidative damage (Liu et al., 2013). As discussed earlier, sugar metabolism plays important roles in maintaining ROS homeostasis. It has also been proposed that there is an interaction between sugar- and ROS-signaling pathways (Bolouri-Moghaddam et al., 2010). Thus, it can be inferred that sugar metabolism may play its signaling role indirectly through interacting with ROS signaling, particularly under stress conditions.

## Roles of other sugar-derived signaling systems in the abiotic stress response

### Trehalose and trehalose-6-phosphate

Trehalose is a non-reducing disaccharide sugar, which is synthesized by trehalose-6-phosphate synthase (TPS) and trehalose-6-phosphate phosphatase (TPP). TPS catalyzes the formation of trehalose-6-phosphate (T6P) from glucose-6-phosphate and UDP-glucose, while TPP is responsible for the dephosphorylation of T6P to produce trehalose (Eastmond et al., 2002). Trehalose is ubiquitous in the biosphere, but only in trace amounts in most plants. Although plenty of evidence indicates that trehalose may play important regulatory roles in plant development and resistance to diverse abiotic stresses (Li et al., 2011), emerging evidence has proposed that T6P, rather than trehalose, is the main active component in trehalose metabolism (Eastmond et al., 2002; Schluepmann et al., 2003; O'Hara et al., 2013). Most previous studies on trehalose metabolism only focus on trehalose, but neglect T6P (Almeida et al., 2005; Cortina and Culiáñez-Macià, 2005; Li et al., 2011). As indicated above, T6P is the precursor of trehalose in the biosynthetic pathway, which has lower content than trehalose in plants and thus is hardly detectable. For example, the concentrations of trehalose and T6P in Arabidopsis seedlings are 26.4 and 0.148 nmol g^−1^ FW, respectively (Van Houtte et al., 2013). This may be the most likely reason why many previous studies did not address T6P.

Trace amount of T6P in most plants implies that T6P may function as a sugar signal. Most recent studies showed decreased T6P level in Arabidopsis by knocking down the expression of *AtTPS1* led to down-regulated expression of flower-triggering genes (e.g., *FT* and *TSF*) and thus a delay in flowering (Wahl et al., 2013). However, sugar availability (sucrose level) increased in the transgenic plants, indicating T6P acts as a signal to regulate the flower initiation of Arabidopsis (Wahl et al., 2013). Arabidopsis TPS (*AtTPS1*) protein interacted with the cell cycle kinase CDKA;1, suggesting the involvement of trehalose metabolism in cell cycle regulation. T6P may also participate in hormone signaling pathways. For example, increased T6P level down-regulated the expression of *auxin/indole-3-acetic acid* (*Aux/IAA*) and *TIR1* in Arabidopsis, two important genes in auxin sensing and signaling (Paul et al., 2010).

A large body of data showed that trehalose metabolism plays important roles in plant resistance to diverse abiotic stresses (Li et al., 2011). For example, the overexpression of the Arabidopsis TPS gene (*AtTPS1*) gene in tobacco increased tolerance to osmotic stress, drought, desiccation, and temperature stresses (Almeida et al., 2005). The introduction of yeast TPS1 gene into tomato enhanced plant tolerance to drought, salt and oxidative stress (Cortina and Culiáñez-Macià, 2005). Recently, native overexpressing of the rice TPS gene (*OsTPS1*) increased the tolerance of rice seedlings to salinity, drought and cold, possibly through up-regulating the expression of some abiotic stress-related genes including *WSI18*, *RAB16C, HSP70*, and *ELIP* (Li et al., 2011). T6P has been proposed to acts as signal molecules to sense carbon availability (O'Hara et al., 2013), a possible mechanism for T6P to regulate plants growth and development in response to abiotic stress. T6P level in plants is positively related to sucrose levels (Schluepmann et al., 2004; Lunn et al., 2006; Martínez-Barajas et al., 2011). Thus, limited sugar availability under various abiotic stresses may reduce the content of T6P. Although no evidence showed there is a direct connection between T6P and the hexokinase-dependent sugar signaling pathway, Schluepmann et al. (2004) suggested there may be a link between T6P and sucrose non-fermenting related kinase-1 (SnRK1), which is known as a inhibitory factor of plant growth (Smeekens et al., 2010). T6P has an inhibitory effect on the activity of SnRK1 (Zhang et al., 2009; Paul et al., 2010). Thus, reduced T6P level under abiotic stresses may lead to an increase in SnRK1 activity and consequently inhibit the biosynthetic processes and plant growth.

### Target of rapamycin

TOR is one member of the family of phosphatidylinositol kinase-related kinases, which is structurally and functionally conserved among organisms and found in nearly all eukaryotes. The TOR signaling has been well studied in fungi and animals (Smeekens et al., 2010) and plays important roles in cell growth and metabolism by integrating the response of cells to growth factors, nutrients, energy, and stress signals (Ahn et al., 2011). Although there is a scarcity of data on TOR in plants, recently it has been proposed to play an important role in plant development through coordinating development with nutrient availability including sugar (Robaglia et al., 2012). For example, AtTOR protein is indispensable for the normal plant and seed development in Arabidopsis (Deprost et al., 2007). Sugar abundance and starvation activate and inhibit the TOR kinase, respectively, followed by up- and down-regulation of energy-consuming related cellular processes, such as mRNA translation and cell proliferation, respectively (Robaglia et al., 2012). Rapamycin can effectively inhibit Arabidopsis TOR kinase activation by glucose and thus retards glucose-mediated root and leaf growth, indicating the central roles of glucose-TOR signaling in plant development (Xiong and Sheen, 2012). These authors further revealed that glucose-TOR signaling is another glucose signaling pathway in parallel with glucose-HXK and plays important roles in reactivation of the cell cycle in quiescent root meristems of Arabidopsis. This glucose-TOR signaling pathway is decoupled from hormone signaling and it was identified that E2Fa transcription factor is the downstream target of the glucose-TOR signaling pathway, which is responsible for the activation of cell cycle (S-phase) genes after its phosphorylation mediated by TOR kinase (Xiong et al., 2013). Sucrose can also activate this signaling pathway, while fructose can not (Xiong et al., 2013).

### SnRKs signaling

Sucrose non-fermenting-1(SNF1)-related protein kinases (SnRKs) is a homolog of the fungal SNF1, which is a global regulator of carbon metabolism in fungi (Coello et al., 2011). Plant SnRKs can be divided into three subgroups: SnRK1s, SnRK2s, and SnRK3s among which SnRK2s and SnRK3s emerged as a result of duplication during plant evolution. It is suggested that SnRK2s and SnRK3s allow plants to link metabolic and stress signaling (Halford and Hey, 2009), which does not occur in mammals and fungi (Coello et al., 2011). This feature gives SnRKs a potential role in regulation of plant responses to stresses. To deal with the sugar limitation (starvation) during stress conditions, plants take measures to limit anabolism and enhance catabolism to save energy and nutrients, in which SnRK act as central regulators (Guérinier et al., 2013). Limited sugar availability activates the activity of SnRKs (Cho et al., 2012), which act as inhibitors of gene expression involved in biosynthetic pathways (Baena-González et al., 2007; Baena-González and Sheen, 2008). At the same time, SnRK1 triggers starch degradation and mobilization under starvation conditions such as darkness (Avila et al., 2012).

In terms of responses to abiotic stresses, loss-of-function of *SOS2*, a SnRK3 gene in Arabidopsis, made plants more sensitive to salt stress, indicating SnRK3 is indispensable for salt tolerance in Arabidopsis (Liu et al., 2000). The knockout or overexpression of SRK2C, an osmotic-stress-activated SnRK2 protein kinase, led to drought-sensitive or tolerant phenotypes, respectively, in Arabidopsis via controlling the expression of stress-related genes (Umezawa et al., 2004). Previous studies showed SnRKs are involved in the ABA signaling pathway, which has been proposed to have a key role in plant tolerance to various abiotic stresses (e.g., salt stress, drought and heat stress) (Coello et al., 2011). For example, the overexpression of *PKABA1*, a SnRK2 gene from barley, mimicked the suppression of ABA to GA-inducible genes in the aleurone layer, indicating a possible role of *PKABA1* in mediating the antagonism between ABA and GA (Gómez-Cadenas et al., 1999). Arabidopsis triple mutant disruptive in three SnRK2s (i.e., SnRK2.2, 2.3, and 2.6) was completely insensitive to ABA treatment (Fujii and Zhu, 2009). SnRK1 has roles in regulation of cell cycle progression in plants. For instance, SnRK1 expression is high in leaf primordia of tomato, but low in meristems (Pien et al., 2001). Most recent studies showed that SnRK1 is involved in the regulation of cell cycle progression in Arabidopsis by controlling the phosphorylation of CDKI p27KIP1 homologs, AtKRP6 and AtKRP7 (Guérinier et al., 2013). The kinase activity of SnRK1 can be inhibited via the phosphorylation by protein kinase AvrPto-dependent Pto-interacting protein3 (Adi3), a suppressor of cell death, indicating SnRK participation in regulation of cell death (Avila et al., 2012).

### bZIPs

The basic leucine-zipper (bZIP) proteins are a large family of multifunctional transcription factors which are characterized by a basic DNA binding region and a leucine-zipper coiled-coil motif in eukaryotes (Reinke et al., 2013). Emerging evidence showed that bZIPs play roles in sugar signaling. It has been proposed that Arabidopsis S1 class bZIPs are transducers of sucrose-specific signals (Weltmeier et al., 2009). At the same time, bZIP11 is also a potential target of SnRK1 (O'Hara et al., 2013).

bZIPs can perceive the variation in sugar status of plants and regulate the profile of gene expression, and thus plant response to internal and external signals (Kang et al., 2010). High sugar availability usually down-regulates the activity of bZIP. For example, sucrose inhibited the translation of *AtbZIP11* mRNA in Arabidopsis (Rook et al., 1998). Glucose repressed the transcription of *AtbZIP1* in Arabidopsis via a HK-dependent pathway (Kang et al., 2010). However, glucose repressed the transcription of *AtbZIP63* in Arabidopsis in an HK-independent way (Matiolli et al., 2011). Furthermore, the full repression of *AtbZIP63* expression by glucose at a high concentration (6%) required the participation of ABA, indicating the involvement of *AtbZIP63* in the glucose-ABA interaction network (Matiolli et al., 2011). Thus, it appears that the mechanism of the bZIP inhibition by sugar is complex and varies with changes in sugar species and levels.

Similar to SnRKs, bZIPs also act as growth inhibitory regulators (Smeekens et al., 2010). For example, the overexpression of *AtbZIP1* and *AtbZIP11* halted the seedling growth of Arabidopsis (Hanson et al., 2008; Kang et al., 2010). The growth inhibition from overexpression of bZIP11 can be relieved by T6P accumulation from trehalose feeding (Schluepmann et al., 2004; Delatte et al., 2011). bZIPs are known to be involved in nutrient (e.g., sugar) and/or stress signaling (Kang et al., 2010; Smeekens et al., 2010; O'Hara et al., 2013) to gain a homeostasis between plant growth and nutrient availability, especially under stress conditions. Plenty of evidence has shown that bZIPs play an important role in the response of plants to various stresses. For example, Arabidopsis seedlings overexpressing a pepper bZIP (*CAbZIP1*) showed enhanced resistance to drought and salt stresses, but the phenotype was dwarfed under optimal condition (Lee et al., 2006), indicating the enhanced stress tolerance may be produced at the cost of normal development. Arabidopsis seedlings expressing soybean bZIP proteins (GmbZIP44, GmbZIP62, or GmbZIP78) exhibited higher tolerance to salt and cold stress, possibly through negative interaction with ABA signaling (Liao et al., 2008). However, rice seedlings overexpressing *OsbZIP52* showed significantly increased sensitivity to cold and drought stress (Liu et al., 2012).

As detailed above, sugar-derived signaling systems (T6P, TOR, SnRK, and bZIP) play important roles in plant responses to abiotic stresses through maintaining the homoeostasis between sugar availability and plant metabolism (Smeekens et al., 2010; Robaglia et al., 2012). Although most of these studies focused on the vegetative stage, the understanding and manipulation of these signaling systems provide new potential options for improvement of seed and fruit set under abiotic stresses including heat and drought.

## A possible regulatory network underlying sugar-mediated control of fruit and seed set

The analyses above allow a possible regulatory network to be formulated about how sucrose metabolism and signaling regulate fruit and seed set under abiotic stresses (Figure 1). Under optimal conditions, sucrose import and its degradation by INV or Sus produce adequate hexoses for development of fruit and seed (or other sink organs). Glucose signaling can facilitate cell division directly or through promoting the response of growth-related hormones (auxin, GA, and cytokinin) and inhibiting senescence-related hormone pathways, hence allowing fruit and seed set (Figure 1A). At the same time, T6P and TORs signaling networks can promote plant growth through up-regulating the expression of anabolism-related genes, whereas SnRKs and bZIP signaling networks with growth inhibitory effects are restrained under optimal conditions. On the other hand, glucose could enter the oxidative pentose phosphate pathway to provide carbon skeletons and energy for biosynthesis of Hsps and non-enzymic antioxidants, thereby preventing oxidative stress and fruit and seed abortion. However, the reverse applies under stress conditions (Figure 1B) where a decreased glucose level could lead to hormonal imbalance, excessive ROS and insufficient Hsps, which may arrest cell division and trigger PCD. Furthermore, sugar limitation inhibits activities of T6P and TOR signaling, but induces activities of SnRKs and bZIPs signaling which can also inhibit cell proliferation through suppressing expression of biosynthetic genes. Together, these biochemical and molecular features could lead to seed and fruit abortion or stunted growth under stress conditions (Figure 1B).

## Conclusion and perspectives

A large body of evidence indicates that sugar metabolism and signaling play important regulatory roles in fruit and seed set and their subsequent development. It is clear that more direct molecular evidence is required to dissect the causal and consequential relationship between changes in sugar metabolism and other biochemical processes and associated phenotype. For instance, although it is well known that glucose metabolism affects hormone levels, it remains to be determined whether the impact is exerted through modulating hormonal sensing and signaling or metabolism and how different hormones coordinate to regulate fruit and seed set under stresses. Second, experimental evidence is still lacking as to whether, and to what extent, biosynthesis of Hsps and maintenance of ROS homeostasis are dependent on sugar metabolism and signaling. Third, previous studies mostly focused on the down-regulation of sugar metabolism, which often has multiple detrimental effects on phenotype and consequently masks the key changes in reproductive development (Boyer and McLaughlin, 2007; Zanor et al., 2009). Little is known about what will happen to fruit and seed development under abiotic stress if key sugar metabolic enzymes, INV and Sus, are up-regulated. Fourth, mounting evidence shows that T6P, TOR, SnRK, and bZIP signaling networks play central roles in plant development through integrating sugar availability and developmental programmes to optimize plant performance. However, no sensor has been identified in perception of sugar status in these networks and the molecular functions and the regulatory mechanisms of them are still largely obscure. Thus, more work needs to be dedicated to this field. Finally, the global analysis of stress responsive regulatory pathways by using new techniques such as RNAseq will help us to understand the signaling cascades by which sugar metabolism and signaling regulate fruit and seed response to heat and drought.

### Conflict of interest statement

The authors declare that the research was conducted in the absence of any commercial or financial relationships that could be construed as a potential conflict of interest.

## References

[B1] Almeida A. M. Villalobos E. Araújo S. S. Leyman B. Van Dijck P. Alfaro-Cardoso L. (2005). Transformation of tobacco with an *Arabidopsis thaliana* gene involved in trehalose biosynthesis increases tolerance to several abiotic stresses. Euphytica 146, 165–176 10.1007/s10681-005-7080-0

[B2] Andersen M. N. Asch F. Wu Y. Jensen C. R. Naested H. Mogensen V. O. (2002). Soluble invertase expression is an early target of drought stress during the critical, abortion sensitive phase of young ovary development in maize. Plant Physiol. 130, 591–604 10.1104/pp.005637 12376627 PMC166589

[B3] Andriunas F. A. Zhang H. M. Xue X. Offler C. E. McCurdy D. W. Patrick J. W. (2012). Reactive oxygen species form part of a regulatory pathway initiating trans-differentiation of epidermal transfer cells in *Vicia faba* cotyledons. J. Exp. Bot. 63, 3617–3630 10.1093/jxb/ers029 22442421 PMC3388844

[B4] Ahn C. S. Han J. A. Lee H. S. Lee S. Pai H. S. (2011). The PP2A regulatory subunit Tap46, a component of the TOR signaling pathway, modulates growth and metabolism in plants. Plant Cell 23, 185–209 10.1105/tpc.110.074005 21216945 PMC3051261

[B5] Avila J. Gregory O. G. Su D. Deeter T. A. Chen S. Silva-Sanchez C. (2012). The β-subunit of the SnRK1 complex is phosphorylated by the plant cell death suppressor Adi3. Plant Physiol. 159, 1277–1290 10.1104/pp.112.198432 22573803 PMC3387709

[B6] Baena-González E. Rolland F. Thevelein J. M. Sheen Jen. (2007). A central integrator of transcription networks in plant stress and energy signalling. Nature 448, 938–942 17671505 10.1038/nature06069

[B7] Baena-González E. Sheen J. (2008). Convergent energy and stress signaling. Trends Plant Sci. 13, 474–482 10.1016/j.tplants.2008.06.006 18701338 PMC3075853

[B8] Baldet P. Hernould M. Laporte F. Mounet F. Just D. Mouras A. (2006). The expression of cell proliferation-related genes in early developing flowers is affected by a fruit load reduction in tomato plants. J. Exp. Bot. 57, 961–970 10.1093/jxb/erj082 16488916

[B9] Barnabás B. Jager K. Feher A. (2008). The effect of drought and heat stress on reproductive processes in cereals. Plant Cell Environ. 31, 11–38 17971069 10.1111/j.1365-3040.2007.01727.x

[B10] Battisti D. S. Naylor R. L. (2009). Historical warnings of future food insecurity with unprecedented seasonal heat. Science 323, 240–244 10.1126/science.1164363 19131626

[B11] Beddington J. (2010). Food security: contributions from science to a new and greener revolution. Philos. Trans. R. Soc. Lond. B Biol. Sci. 365, 61–71 10.1098/rstb.2009.0201 20008386 PMC2842707

[B12] Beere H. M. (2004). “The stress of dying”: the role of heat shock proteins in the regulation of apoptosis. J. Cell Sci. 117, 2641–2651 10.1242/jcs.01284 15169835

[B13] Bienert G. P. Møller A. L. B. Kristiansen K. A. Schulz A. Møller I. M. Schjoerring J. K. (2007). Specific aquaporins facilitate the diffusion of hydrogen peroxide across membranes. J. Biol. Chem. 282, 1183–1192 10.1074/jbc.M603761200 17105724

[B14] Blanke M. M. Lenz F. (1989). Fruit photosynthesis. Plant Cell Environ. 12, 31–46 10.1111/j.1365-3040.1989.tb01914.x

[B15] Bolouri-Moghaddam M. Le Roy K. Xiang L. Rolland F. Van den Ende W. (2010). Sugar signalling and antioxidant network connections in plant cells. FEBS J. 277, 2022–2037 10.1111/j.1742-4658.2010.07633.x 20412056

[B16] Boyer J. S. McLaughlin J. E. (2007). Functional reversion to identify controlling genes in multigenic responses: analysis of floral abortion. J. Exp. Bot. 58, 267–277 10.1093/jxb/erl177 17105969

[B17] Chao Q. Rothenberg M. Solano R. Roman G. Terzaghi W. Ecker J. R. (1997). Activation of the ethylene gas response pathway in *Arabidopsis* by the nuclear protein ETHYLENE-INSENSITIVE 3 and related proteins. Cell 89, 1133–1144 10.1016/S0092-867480300-1 9215635

[B19] Cho Y. H. Hong J. W. Kim E. C. Yoo S. D. (2012). Regulatory functions of SnRK1 in stress-responsive gene expression and in plant growth and development. Plant Physiol. 158, 1955–1964 10.1104/pp.111.189829 22232383 PMC3320198

[B18] Cho Y. H. Yoo S. D. (2011). Signaling role of fructose mediated by FINS1/FBP in *Arabidopsis thaliana*. PLoS Genet. 7:1001263 10.1371/journal.pgen.1001263 21253566 PMC3017112

[B20] Coello P. Hey S. J. Halford N. G. (2011). The sucrose non-fermenting-1-related (SnRK) family of protein kinases: potential for manipulation to improve stress tolerance and increase yield. J. Exp. Bot. 62, 883–893 10.1093/jxb/erq331 20974737

[B21] Cortina C. Culiáñez-Macià F. A. (2005). Tomato abiotic stress enhanced tolerance by trehalose biosynthesis. Plant Sci. 169, 75–82

[B22] Couée I. Sulmon C. Gouesbet G. El Amrani A. (2006). An involvement of soluble sugars in reactive oxygen species balance and responses to oxidative stress in plants. J. Exp. Bot. 57, 449–459 10.1093/jxb/erj027 16397003

[B23] Dat J. F. Lopez-Delgado H. Foyer C. H. Scott I. M. (1998). Parallel changes in H_2_O_2_ and catalase during thermotolerance induced by salicylic acid or heat acclimation in mustard seedlings. Plant Physiol. 116, 1351–1357 10.1104/pp.116.4.1351 9536052 PMC35042

[B24] Davies P. J. (2010). The plant hormones: their nature, occurrence, and functions in Plant Hormones: Biosynthesis, Signal Transduction, Action! ed Davies P. (Dordrecht: Springer Press), 1–15

[B25] Delatte T. L. Sedijani P. Kondou Y. Matsui M. de Jong G. J. Somsen G. W. (2011). Growth arrest by trehalose-6-phosphate: an astonishing case of primary metabolite control over growth by way of the SnRK1 signaling pathway. Plant Physiol. 157, 160–174 10.1104/pp.111.180422 21753116 PMC3165867

[B26] Deprost D. Yao L. Sormani R. Moreau M. Leterreux G. Nicolai M. (2007). The *Arabidopsis* TOR kinase links plant growth, yield, stress resistance and mRNA translation. EMBO Rep. 8, 864–870 10.1038/sj.embor.7401043 17721444 PMC1973950

[B27] Dewitte W. Murray J. A. H. (2003). The plant cell cycle. Annu. Rev. Plant Biol. 54, 235–264 10.1146/annurev.arplant.54.031902.134836 14502991

[B28] Dorcey E. Urbez C. Blázquez M. A. Carbonell J. Perez-Amador M. A. (2009). Fertilization-dependent auxin response in ovules triggers fruit development through the modulation of gibberellin metabolism in *Arabidopsis*. Plant J. 58, 318–332 10.1111/j.1365-313X.2008.03781.x 19207215

[B29] Döös B. R. (2002). Population growth and loss of arable land. Glob. Environ. Change 12, 303–311 10.1016/S0959-378000043-2

[B30] Dugas D. V. Bartel B. (2008). Sucrose induction of *Arabidopsis* miR398 represses two Cu/Zn superoxide dismutases. Plant Mol. Biol. 67, 403–417 10.1007/s11103-008-9329-1 18392778

[B31] Eastmond P. J. Van Dijken A. J. H. Spielman M. Kerr A. Tissier A. Dickinson H. G. (2002). Trehalose-6-phosphate synthase 1, which catalyses the first step in trehalose synthesis, is essential for *Arabidopsis* embryo maturation. Plant J. 29, 225–235 10.1046/j.1365-313x.2002.01220.x 11851922

[B32] Egli D. B. (2010). SOYPOD: a model of fruit set in soybean. Agron. J. 102, 39–47 10.2134/agronj2009.0222

[B33] Ehness R. Roitsch T. (1997). Co-ordinated induction of mRNAs for extracellular invertase and a glucose transporter in *Chenopodium rubrum* by cytokinins. Plant J. 11, 539–548 10.1046/j.1365-313X.1997.11030539.x 9107040

[B34] Fedoroff N. V. Battisti D. S. Beachy R. N. Cooper P. J. M. Fischhoff D. A. Hodges C. N. (2010). Radically rethinking agriculture for the 21st century. Science 327, 833–834 10.1126/science.1186834 20150494 PMC3137512

[B35] Finka A. Mattoo R. U. H. Goloubinoff P. (2011). Meta-analysis of heat- and chemically upregulated chaperone genes in plant and human cells. Cell Stress Chaperones 16, 15–31 10.1007/s12192-010-0216-8 20694844 PMC3024091

[B36] Finka A. Quendet A. F. H. Maathuis F. J. M. Saidi Y. Goloubinoff P. (2012). Plasma membrane cyclic nucleotide gated calcium channels control land plant thermal sensing and acquired thermotolerance. Plant Cell 24, 3333–3348 10.1105/tpc.112.095844 22904147 PMC3462635

[B37] Foulkes M. J. Slafer G. A. Davies W. J. Berry P. M. Sylvester-Bradley R. Martre P. (2011). Raising yield potential of wheat. III. optimizing partitioning to grain while maintaining lodging resistance. J. Exp. Bot. 62, 469–486 10.1093/jxb/erq300 20952627

[B38] Foyer C. H. Noctor G. (2005). Oxidant and antioxidant signaling in plants: a re-evaluation of the concept of oxidative stress in a physiological context. Plant Cell Environ. 28, 1056–1071 10.1111/j.1365-3040.2005.01327.x

[B39] Fujii H. Zhu J. K. (2009). *Arabidopsis* mutant deficient in 3 abscisic acid-activated protein kinases reveals critical roles in growth, reproduction, and stress. Proc. Natl. Acad. Sci. U.S.A. 1106, 8380–8385 10.1073/pnas.0903144106 19420218 PMC2688869

[B40] Garrity D. P. Akinnifesi F. K. Ajayi O. C. Weldesemayat S. G. Mowo J. G. Kalinganire A. (2010). Evergreen agriculture: a robust approach to sustainable food security in africa. Food Secur. 2, 197–214 10.1007/s12571-010-0070-7

[B41] Ghiglione H. O. Gonzalez F. G. Serrago R. Maldonado S. B. Chilcott C. Curá J. A. (2008). Autophagy regulated by day length determines the number of fertile florets in wheat. Plant J. 55, 1010–1024 10.1111/j.1365-313X.2008.03570.x 18547393

[B42] Gillaspy G. Ben-David H. Gruissem W. (1993). Fruits: a developmental perspective. Plant Cell 5, 1439–1451 12271039 10.1105/tpc.5.10.1439 PMC160374

[B43] Godfray H. C. J. Beddington J. R. Crute I. R. Haddad L. Lawrence D. Muir J. F. (2010). Food security: the challenge of feeding 9 billion people. Science 327, 812–818 10.1126/science.1185383 20110467

[B44] Gómez-Cadenas A. Verhey S. D. Holappa L. D. Shen Q. Ho T.-H. D. Walker-Simmons M. K. (1999). An abscisic acid-induced protein kinase, PKABA1, mediates abscisic acid-suppressed gene expression in barley aleurone layers. Proc. Natl. Acad. Sci. U.S.A. 96, 1767–1772 10.1073/pnas.96.4.1767 9990099 PMC15589

[B45] Guérinier T. Millan L. Crozet P. Oury C. Rey F. Valot B. (2013). Phosphorylation of p27KIP1 homologs KRP6 and 7 by SNF1-related protein kinase-1 links plant energy homeostasis and cell proliferation. Plant J. 10.1111/tpj.12218 23617622

[B46] Halford N. G. Hey S. J. (2009). SNF1-related protein kinases (SnRKs) act within an intricate network that links metabolic and stress signalling in plants. Biochem. J. 419, 247–259 10.1042/BJ20082408 19309312

[B47] Hanson J. Hanssen M. Wiese A. Hendriks M. M. Smeekens S. (2008). The sucrose regulated transcription factor bZIP11 affects amino acid metabolism by regulating the expression of ASPARAGINE SYNTHETASE1 and PROLINE DEHYDROGENASE2. Plant J. 53, 935–949 10.1111/j.1365-313X.2007.03385.x 18088315

[B48] Hedhly A. Hormaza J. I. Herrero M. (2008). Global warming and plant sexual reproduction. Trends Plant Sci. 14, 30–36 10.1016/j.tplants.2008.11.001 19062328

[B49] Ho L. C. (1988). Metabolism and compartmentation of imported sugars in sink organs in relation to sink strength. Annu. Rev. Plant Physiol. Plant Mol. Biol. 39, 355–378 10.1146/annurev.pp.39.060188.002035

[B50] Horváth I. Glatz A. Varvasovszki V. Török Z. Páli T. Balogh G. (1998). Membrane physical state controls the signaling mechanism of the heat shock response in *Synechocystis* PCC 6803: Identification of *hsp17* as a “fluidity gene”. Proc. Natl. Acad. Sci. U.S.A. 95, 3513–3518 10.1073/pnas.95.7.3513 9520397 PMC19867

[B51] Ji X. Dong B. Shiran B. Talbot M. J. Edlington J. E. Hughes T. (2011). Control of abscisic acid catabolism and abscisic acid homeostasis is important for reproductive stage stress tolerance in cereals. Plant Physiol. 156, 647–662 10.1104/pp.111.176164 21502188 PMC3177265

[B52] Jia H. Wang Y. Sun M. Li B. Han Y. Zhao Y. (2013). Sucrose functions as a signal involved in the regulation of strawberry fruit development and ripening. New Phytol. 198, 453–465 10.1111/nph.12176 23425297

[B53] Jin Y. Ni D. A. Ruan Y. L. (2009). Posttranslational elevation of cell wall invertase activity by silencing its inhibitor in tomato delays leaf senescence and increases seed weight and fruit hexose level. Plant Cell 21, 2072–2089 10.1105/tpc.108.063719 19574437 PMC2729613

[B54] Kakumanu A. Ambavaram M. M. R. Klumas C. Krishnan A. Batlang U. Myers E. (2012). Effects of drought on gene expression in maize reproductive and leaf meristem tissue revealed by RNA-Seq. Plant Physiol. 160, 846–867 10.1104/pp.112.200444 22837360 PMC3461560

[B55] Kang S. G. Price J. Lin P. C. Hong J. C. Jang J. C. (2010). The *Arabidopsis* bZIP1 transcription factor is involved in sugar signaling, protein networking, and DNA binding. Mol. Plant 3, 361–373 10.1093/mp/ssp115 20080816

[B56] Kim J. Y. Mahé A. Brangeon J. Prioul J. L. (2000). A maize vacuolar invertase, *IVR*_2_, is induced by water stress: organ/tissue specificity and diurnal modulation of expression. Plant Physiol. 124, 71–84 10.1104/pp.124.1.71 10982423 PMC59123

[B57] Koch K. E. (2004). Sucrose metabolism: regulatory mechanisms and pivotal roles in sugar sensing and plant development. Curr. Opin. Plant Biol. 7, 235–246 10.1016/j.pbi.2004.03.014 15134743

[B58] Kovtun Y. Chiu W. L. Tena G. Sheen J. (2000). Functional analysis of oxidative stress-activated mitogen-activated protein kinase cascade in plants. Proc. Natl. Acad. Sci. U.S.A. 97, 2940–2945 10.1073/pnas.97.6.2940 10717008 PMC16034

[B59] Laloi C. Apel K. Danon A. (2004). Reactive oxygen signaling: the latest news. Curr. Opin. Plant Biol. 7, 323–328 10.1016/j.pbi.2004.03.005 15134754

[B60] Lara M. E. B. Garcia M. C. G. Fatima T. Ehneb R. Lee T. K. Proels R. (2004). Extracellular invertase is an essential component of cytokinin-mediated delay of senescence. Plant Cell 16, 1276–1287 10.1105/tpc.018929 15100396 PMC423215

[B61] LeClere S. Schmelz E. A. Chourey P. S. (2010). Sugar levels regulate tryptophan-dependent auxin biosynthesis in developing maize kernels. Plant Physiol. 153, 306–318 10.1104/pp.110.155226 20237017 PMC2862422

[B62] Lee S. C. Choi H. W. Hwang I. S. Choi D. S. Hwang B. K. (2006). Functional roles of the pepper pathogen-induced bZIP transcription factor, CAbZIP1, in enhanced resistance to pathogen infection and environmental stresses. Planta 224, 1209–1225 10.1007/s00425-006-0302-4 16718483

[B63] Li H. W. Zang B. S. Deng X. W. Wang X. P. (2011). Overexpression of the trehalose-6-phosphate synthase gene *OsTPS1* enhances abiotic stress tolerance in rice. Planta 234, 1007–1018 10.1007/s00425-011-1458-0 21698458

[B64] Li Z. Palmer W. M. Martin A. P. Wang R. Rainsford F. Jin Y. (2012). High invertase activity in tomato reproductive organs correlates with enhanced sucrose import into, and heat tolerance of, young fruit. J. Exp. Bot. 63, 1155–1166 10.1093/jxb/err329 22105847 PMC3276082

[B65] Liao Y. Zou H. F. Wei W. Hao Y. J. Tian A. G. Huang J. (2008). Soybean GmbZIP44, GmbZIP62 and GmbZIP78 genes function as negative regulator of ABA signaling and confer salt and freezing tolerance in transgenic *Arabidopsis*. Planta 228, 225–240 18365246 10.1007/s00425-008-0731-3

[B66] Liu J. Ishitani M. Halfter U. Kim C. S. Shu J. K. (2000). The *Arabidopsis thaliana SOS2* gene encodes a protein kinase that is required for salt tolerance. Proc. Natl. Acad. Sci. U.S.A. 97, 3730–3734 10725382 10.1073/pnas.060034197 PMC16308

[B68] Liu C. Wu Y. Wang X. (2012). bZIP transcription factor OsbZIP52/RISBZ5: a potential negative regulator of cold and drought stress response in rice. Planta 235, 1157–1169 22189955 10.1007/s00425-011-1564-z

[B67] Liu J. Wang X. Hu Y. Hu W. Bi Y. (2013). Glucose-6-phosphate dehydrogenase plays a pivotal role in tolerance to drought stress in soybean roots. Plant Cell Rep. 32, 415–429 10.1007/s00299-012-1374-1 23233130

[B69] Liu F. Jensen C. R. Andersen M. N. (2004). Drought stress effect on carbohydrate concentration in soybean leaves and pods during early reproductive development, its implication in altering pod set. Field Crops Res. 86, 1–13 10.1016/S0378-429000165-5

[B70] Lunn J. E. Feil R. Hendriks J. H. M. Gibon Y. Morcuende R. Osuna D. (2006). Sugar-induced increases in trehalose 6-phosphate are correlated with redox activation of ADPglucose pyrophosphorylase and higher rates of starch synthesis in *Arabidopsis thaliana*. Biochem. J. 397(Pt 1), 139–148 16551270 10.1042/BJ20060083 PMC1479759

[B71] Lytovchenko A. EickmeierPons C. Osorio S. Szecowka M. Lehmberg K. Arrivault S. (2011). Tomato fruit photosynthesis is seemingly unimportant in primary metabolism and ripening but plays a considerable role in seed development. Plant Physiol. 157, 1650–1663 10.1104/pp.111.186874 21972266 PMC3327185

[B72] Macgregor D. R. Deak K. I. Ingram P. A. Malamy J. E. (2008). Root system architecture in *Arabidopsis* grown in culture is regulated by sucrose uptake in the aerial tissues. Plant Cell 20, 2643–2660 10.1105/tpc.107.055475 18952782 PMC2590740

[B73] Malik M. K. Slovin J. P. Hwang C. H. Zimmerman J. L. (1999). Modified expression of a carrot small heat shock protein gene, *hsp17.7*, results in increased or decreased thermotolerance double dagger. Plant J. 20, 89–99 10.1046/j.1365-313X.1999.00581.x 10571868

[B74] Martínez-Barajas E. Delatte T. Schluepmann H. de Jong G. J. Somsen G. W. Nunes C. (2011). Wheat grain development is characterized by remarkable trehalose 6-phosphate accumulation pregrain filling: tissue distribution and relationship to SNF1-related protein kinase1 activity. Plant Physiol. 156, 373–381 10.1104/pp.111.174524 21402798 PMC3091070

[B75] Matiolli C. C. Tomaz J. P. Duarte G. T. Prado F. M. Del Bem L. E. V. Silveira A. B. (2011). The *Arabidopsis* bZIP gene AtbZIP63 is a sensitive integrator of transient abscisic acid and glucose signals. Plant Physiol. 157, 692–705 10.1104/pp.111.181743 21844310 PMC3192551

[B76] McLaughlin J. E. Boyer J. S. (2004a). Glucose localisation in maize ovaries when kernel number decreases at low water potential and sucrose is fed to the stems. Ann. Bot. 94, 75–86 15159218 10.1093/aob/mch123 PMC4242379

[B77] McLaughlin J. E. Boyer J. S. (2004b). Sugar-responsive gene expression, invertase activity, and senescence in aborting maize ovaries at low water potentials. Ann. Bot. 94, 675–689 15355866 10.1093/aob/mch193 PMC4242214

[B78] Miller M. E. Chourey P. S. (1992). The maize invertase-deficient *minature-1* seed mutation is associated with aberrant pedicel and endosperm development. Plant Cell 4, 297–305 12297647 10.1105/tpc.4.3.297 PMC160130

[B79] Mishra B. S. Singh M. Aggrawal P. Laxmi A. (2009). Glucose and auxin signalling interaction in controlling *Arabidopsis thaliana* seedlings root growth and development. PLoS ONE 4:e4502 10.1371/journal.pone.0004502 19223973 PMC2637607

[B80] Mittler R. (2002). Oxidative stress, antioxidants and stress tolerance. Trends Plant Sci. 7, 405–410 10.1016/S1360-138502312-9 12234732

[B81] Mittler R. Blumwald E. (2010). Genetic engineering for modern agriculture: challenges and perspectives. Annu. Rev. Plant Biol. 61, 443–462 10.1146/annurev-arplant-042809-112116 20192746

[B82] Mittler R. Finka A. Goloubinoff P. (2012). How do plants feel the heat. Trends Biochem. Sci. 37, 118–125 22236506 10.1016/j.tibs.2011.11.007

[B83] Moore B. Zhou L. Rolland F. Hall Q. Cheng W. H. Liu Y. X. (2003). Role of the *Arabidopsis* glucose sensor HXK1 in nutrient, light, and hormonal signaling. Science 300, 332–336 10.1126/science.1080585 12690200

[B84] Munné-Bosch S. Queval G. Foyer C. H. (2013). The impact of global change factors on redox signaling underpinning stress tolerance. Plant Physiol. 161, 5–19 10.1104/pp.112.205690 23151347 PMC3532280

[B85] O'Hara L. E. Paul M. J. Wingler A. (2013). How do sugars regulate plant growth and development. New insight into the role of trehalose-6-phosphate. Mol. Plant. 6, 261–274 10.1093/mp/sss120 23100484

[B86] Oktyabrsky O. N. Smirnova G. V. (2007). Redox regulation of cellular functions. Biochemistry (Mosc.) 72, 132–145 10.1134/S0006297907020022 17367290

[B87] Oliver S. N. Dennis E. S. Dolferus R. (2007). ABA regulates apoplastic sugar transport and is a potential signal for cold-induced pollen sterility in rice. Plant Cell Physiol. 48, 1319–1330 10.1093/pcp/pcm100 17693452

[B88] Patrick J. W. Stoddard F. L. (2010). Physiology of flowering and grain filling in faba bean. Field Crops Res. 115, 234–242 10.1016/j.fcr.2009.06.005

[B89] Paul M. J. Jhurreea D. Zhang Y. Primavesi L. F. Delatte T. Schluepmann H. (2010). Up-regulation of biosynthetic processes associated with growth by trehalose 6-phosphate. Plant Signal. Behav. 5, 1–7 10.4161/psb.5.4.10792 20139731 PMC2958589

[B90] Pien S. Wyrzykowska J. Fleming A. J. (2001). Novel marker genes for early leaf development indicate spatial regulation of carbohydrate metabolism within the apical meristem. Plant J. 25, 663–674 10.1046/j.1365-313x.2001.01002.x 11319033

[B91] Pinheiro C. António C. Ortuño M. F. Dobrev P. I. Hartung W. Thomas-Oates J. (2011). Initial water deficit effects on *Lupinus albus* photosynthetic performance, carbon metabolism, and hormonal balance: metabolic reorganization prior to early stress responses. J. Exp. Bot. 62, 4965–4974 21772019 10.1093/jxb/err194

[B92] Queitsch C. Hong S. W. Vierling E. Lindquist S. (2000). Heat shock protein 101 plays a crucial role in thermotolerance in *Arabidopsis*. Plant Cell 12, 479–492 10760238 10.1105/tpc.12.4.479 PMC139847

[B93] Rampino P. Mita G. Fasano P. Borrelli G. M. Aprile A. Dalessandro G. (2012). Novel durum wheat genes up-regulated in response to a combination of heat and drought stress. Plant Physiol. Biochem. 56, 72–78 10.1016/j.plaphy.2012.04.006 22609457

[B94] Reinke A. W. Baek J. Ashenberg O. Keating A. E. (2013). Networks of bZIP protein-protein interactions diversified over a billion years of evolution. Science 340, 730–734 23661758 10.1126/science.1233465 PMC4115154

[B95] Robaglia C. Thomas M. Meyer C. (2012). Sensing nutrient and energy status by SnRK1 and TOR kinases. Curr. Opin. Plant Biol. 15, 301–307 10.1016/j.pbi.2012.01.012 22305521

[B96] Rook F. Gerrits N. Kortstee A. vanKampen M. Borrias M. Weisbeek P. (1998). Sucrose-specific signalling represses translation of the *Arabidopsis* ATB2 bZIP transcription factor gene. Plant J. 15, 253–263 10.1046/j.1365-313X.1998.00205.x 9721683

[B97] Ross-Ibarra J. Morrell P. L. Gaut B. S. (2007). Plant domestication, a unique opportunity to identify the genetic basis of adaptation. Proc. Natl. Acad. Sci. U.S.A. 104, 8641–8648 10.1073/pnas.0700643104 17494757 PMC1876441

[B98] Ruan Y. L. Jin Y. Li G. J. Yang Y. J. Boyer J. S. (2010). Sugar input, metabolism and signaling mediated by invertase: roles in development, yield potential and response to drought and heat. Mol. Plant 3, 942–955 10.1093/mp/ssq044 20729475

[B99] Ruan Y. L. (2012). Signaling role of sucrose metabolism in development. Mol. Plant 5, 763–765 10.1093/mp/sss046 22532605

[B100] Ruan Y. L. Patrick J. W. Bouzayen M. Osorio S. Fernie A. R. (2012). Molecular regulation of seed and fruit set. Trends Plant Sci. 17, 656–665 10.1016/j.tplants.2012.06.005 22776090

[B101] Sairanen I. Novák O. Pìnèík A. Ikeda Y. Jones B. Sandberg G. (2012). Soluble carbohydrates regulate auxin biosynthesis via PIF proteins in *Arabidopsis*. Plant Cell 24, 4907–4916 10.1105/tpc.112.104794 23209113 PMC3556965

[B102] Sangwan V. Orvar B. L. Beyerly J. Hirt H. Dhindsa R. S. (2002). Opposite changes in membrane fluidity mimic cold and heat stress activation of distinct plant MAP kinase pathways. Plant J. 31, 629–638 10.1046/j.1365-313X.2002.01384.x 12207652

[B103] Schluepmann H. Pellny T. van Dijken A. Smeekens S. Paul M. (2003). Trehalose 6-phosphate is indispensible for carbohydrate utilization and growth in *Arabidopsis thaliana*. Proc. Natl. Acad. Sci. U.S.A. 100, 6849–6854 12748379 10.1073/pnas.1132018100 PMC164535

[B104] Schluepmann H. van Dijken A. Aghdasi M. Wobbes B. Paul M. Smeekens S. (2004). Trehalose mediated growth inhibition of *Arabidopsis* seedlings is due to trehalose-6-phosphate accumulation. Plant Physiol. 135, 879–890 10.1104/pp.104.039503 15181209 PMC514123

[B105] Setter T. L. Yan J. Warburton M. Ribaut J. M. Xu Y. Sawkins M. (2011). Genetic association mapping identifies single nucleotide polymorphisms in genes that affect abscisic acid levels in maize floral tissues during drought. J. Exp. Bot. 62, 701–716 10.1093/jxb/erq308 21084430 PMC3003815

[B106] Sharma S. K. De los Rios P. Christen P. Lustig A. Goloubinoff P. (2010). The kinetic parameters and energy cost of the Hsp70 chaperone as a polypeptide unfoldase. Nat. Chem. Biol. 6, 914–920 10.1038/nchembio.455 20953191

[B107] Sinkevich M. S. Naraykina N. V. Trunova T. I. (2010). Involvement of sugars in the antioxidant defense against paraquat-induced oxidative stress in potato transformed with yeast invertase gene. Doklady Biol. Sci. 434, 338–340 10.1134/S0012496610050133 20963659

[B108] Smeekens S. Ma J. Hanson J. Rolland F. (2010). Sugar signals and molecular networks controlling plant growth. Curr. Opin. Plant Biol. 13, 274–279 10.1016/j.pbi.2009.12.002 20056477

[B109] Snider J. L. Oosterhuis D. M. Loka D. A. Kawakami E. M. (2011). High temperature limits *in vivo* pollen tube growth rates by altering diurnal carbohydrate balance in field-grown *Gossypium hirsutum* pistils. J. Plant Physiol. 168, 1168–1175 10.1016/j.jplph.2010.12.011 21256621

[B110] Solomon B. D. (2010). Biofuels and sustainability. Ann. N.Y. Acad. Sci. 1185, 119–134 10.1111/j.1749-6632.2009.05279.x 20146765

[B111] Sturm A. (1999). Invertases: primary structures, functions and roles in plant development and sucrose partitioning. Plant Physiol. 121, 1–7 10.1104/pp.121.1.1 10482654 PMC1539224

[B112] Sturm A. Tang G. Q. (1999). The sucrose-cleaving enzymes of plants are crucial for development, growth and carbon partitioning. Trends Plant Sci. 4, 401–407 10.1016/S1360-138501470-3 10498964

[B113] Sunkar R. Kapoor A. Zhu J. K. (2006). Posttranscriptional induction of two Cu/Zn superoxide dismutase genes in *Arabidopsis* is mediated by downregulation of miR398 and important for oxidative stress tolerance. Plant Cell 18, 2051–2065 10.1105/tpc.106.041673 16861386 PMC1533975

[B114] Suwa R. Hakata H. Hara H. El-Shemy H. A. Adu-Gyamfi J. J. Nguyen N. T. (2010). High temperature effects on photosynthate partitioning and sugar metabolism during ear expansion in maize (*Zea mays* L.) genotypes. Plant Physiol. Biochem. 48, 124–130 10.1016/j.plaphy.2009.12.010 20106675

[B115] Thakur P. Kumar S. Malik J. A. Berger J. D. Nayyar H. (2010). Cold stress effects on reproductive development in grain crops: an overview. Environ. Exp. Bot. 67, 429–443 10.1016/j.envexpbot.2009.09.004

[B116] Tognetti J. A. Pontis H. G. Martínez-Noël G. M. A. (2013). Sucrose signaling in plants: a world yet to be explored. Plant Signal. Behav. 8:e23316 10.4161/psb.23316 23333971 PMC3676498

[B117] Umezawa T. Yoshida R. Maruyama K. Yamaguchi-Shinozaki K. Shinozaki K. (2004). SRK2C, a SNF1-related protein kinase 2, improves drought tolerance by controlling stress-response gene expression in *Arabidopsis thaliana*. Proc. Natl. Acad. Sci. U.S.A 101, 17306–17311 10.1073/pnas.0407758101 15561775 PMC535404

[B118] Vacca R. A. de Pinto M. C. Valenti D. Passarella S. Marra E. De Gara L. (2004). Production of reactive oxygen species, alteration of cytosolic ascorbate peroxidase, and impairment of mitochondrial metabolism are early events in heat shock-induced programmed cell death in tobacco Bright-Yellow 2 cells. Plant Physiol. 134, 1100–1112 10.1104/pp.103.035956 15020761 PMC389934

[B119] Van Breusegem F. Bailey-Serres J. Mittler R. (2008). Unraveling the tapestry of networks involving reactive oxygen species in plants. Plant Physiol. 147, 978–984 10.1104/pp.108.122325 18612075 PMC2442543

[B120] Van den Ende W. Valluru R. (2009). Sucrose, sucrosyl oligosaccharides, and oxidative stress: scavenging and salvaging. J. Exp. Bot. 60, 9–18 10.1093/jxb/ern297 19036839

[B121] Van Houtte H. Vandesteene L. López-Galvis L. Lemmens L. Kissel E. Carpentier S. (2013). Overexpression of the trehalase gene AtTRE1 leads to increased drought stress tolerance in *Arabidopsis* and is involved in abscisic acid-induced stomatal closure. Plant Physiol. 161, 1158–1171 10.1104/pp.112.211391 23341362 PMC3585587

[B122] Vilhar B. Kladnik A. Blejec A. Chourey P. S. Dermastia M. (2002). Cytometrical evidence that the loss of seed weight in the miniature1 seed mutant of maize is associated with reduced mitotic activity in the developing endosperm. Plant Physiol. 129, 23–30 10.1104/pp.001826 12011334 PMC1540223

[B123] Volkov R. A. Panchuk I. I. Mullineaux P. M. Schöffl F. (2006). Heat stress-induced H_2_O_2_ is required for effective expression of heat shock genes in *Arabidopsis*. Plant Mol. Biol. 61, 733–746 10.1007/s11103-006-0045-4 16897488

[B124] Wahl V. Ponnu J. Schlereth A. Stéphanie A. Langenecker T. Franke A. (2013). Regulation of flowering by trehalose-6-phosphate signaling in *Arabidopsis thaliana*. Science 339, 704–707 10.1126/science.1230406 23393265

[B125] Wang E. Wang J. Zhu X. Hao W. Wang L. Li Q. (2008). Control of rice grain-filling and yield by a gene with a potential signature of domestication. Nat. Genet. 40, 1370–1374 10.1038/ng.220 18820698

[B126] Wang H. Schauer N. Usadel B. Frasse P. Zouine M. Hernould M. (2009). Regulatory features underlying pollination-dependent and -independent tomato fruit set revealed by transcript and primary metabolite profiling. Plant Cell 21, 1428–1452 10.1105/tpc.108.060830 19435935 PMC2700536

[B127] Weber H. Borisjuk L. Wobus U. (1996). Controlling seed development and seed size in *Vicia faba*: a role for seed coat-associated invertases and carbohydrate state. Plant J. 10, 823–834

[B128] Weber H. Borisjuk L. Wobus U. (2005). Molecular physiology of legume seed development. Annu. Rev. Plant Biol. 56, 253–279 10.1146/annurev.arplant.56.032604.144201 15862096

[B129] Weil M. Rausch T. (1990). Cell wall invertase in tobacco crown gall cells: enzyme properties and regulation by auxin. Plant Physiol. 94, 1575–1581 10.1104/pp.94.4.1575 16667892 PMC1077423

[B130] Weiss Y. G. Bromberg Z. Raj N. Raphael J. Goloubinoff P. Ben-Neriah Y. (2007). Enhanced heat shock protein 70 expression alters proteasomal degradation of IkappaB kinase in experimental acute respiratory distress syndrome. Crit. Care Med. 35, 2128–2138 10.1097/01.CCM.0000278915.78030.74 17855826

[B131] Weltmeier F. Rahmani F. Ehlert A. Dietrich K. Schutze K. Wang X. (2009). Expression patterns within the *Arabidopsis* C/S1 bZIP transcription factor network: availability of heterodimerization partners controls gene expression during stress response and development. Plant Mol. Biol. 69, 107–119 10.1007/s11103-008-9410-9 18841482 PMC2709229

[B132] Wind J. Smeekens S. Hanson J. (2010). Sucrose: metabolite and signaling molecule. Phytochemistry 71, 1610–1614 10.1016/j.phytochem.2010.07.007 20696445

[B133] Xiang L. Li Y. Rolland F. Van den Ende W. (2011). Neutral invertase, hexokinase and mitochondrial ROS homeostasis: emerging links between sugar metabolism, sugar signaling and ascorbate synthesis. Plant Signal. Behav. 6, 1567–1573 10.4161/psb.6.10.17036 21918379 PMC3256386

[B134] Xiong Y. McCormack M. Li L. Sheen J. (2013). Glucose-TOR signalling reprograms the transcriptome and activates meristems. Nature 496, 181–186 23542588 10.1038/nature12030 PMC4140196

[B135] Xiong Y. Sheen J. (2012). Rapamycin and glucose-target of rapamycin (TOR) protein signaling in plants. J. Biol. Chem. 287, 2836–2842 10.1074/jbc.M111.300749 22134914 PMC3268441

[B136] Xu S. Brill E. Llewellyn D. Furbank R. T. Ruan Y. L. (2012). Over-expression of a potato sucrose synthase gene in cotton accelerates leaf expansion, reduces seed abortion and enhances fiber production. Mol. Plant 5, 430–441 10.1093/mp/ssr090 22115917

[B137] Yanagisawa S. Yoo S. D. Sheen J. (2003). Differential regulation of EIN3 stability by glucose and ethylene signalling in plants. Nature 425, 521–525 10.1038/nature01984 14523448

[B138] Young L. W. Wilen R. W. Bonham-Smith P. C. (2004). High temperature stress of *Brassica napus* during flowering reduces micro- and megagametophyte fertility, induces fruit abortion, and disrupts seed production. J. Exp. Bot. 55, 485–495 10.1093/jxb/erh038 14739270

[B139] Zanor M. I. Osorio S. Nunes-Nesi A. Carrari F. Lohse M. Usadel B. (2009). RNA interference of LIN5 in tomato confirms its role in controlling brix content, uncovers the influence of sugars on the levels of fruit hormones and demonstrates the importance of sucrose cleavage for normal fruit development and fertility. Plant Physiol. 150, 1204–1218 10.1104/pp.109.136598 19439574 PMC2705052

[B140a] Zhang Y. Primavesi L. F. Jhurreea D. Andralojc P. J. Mitchell R. A. C. Powers S. J. (2009). Inhibition of Snf1- related protein kinase (SnRK1) activity and regulation of metabolic pathways by trehalose 6-phosphate. Plant Physiol. 149, 1860–1871 10.1104/pp.108.133934 19193861 PMC2663748

[B140] Zinn K. E. Tunc-Ozdemir M. Harper J. F. (2010). Temperature stress and plant sexual reproduction: uncovering the weakest links. J. Exp. Bot. 61, 1959–1968 10.1093/jxb/erq053 20351019 PMC2917059

[B141] Zinselmeier C. Jeong B. R. Boyer J. S. (1999). Starch and the control of kernel number in maize at low water potentials. Plant Physiol. 121, 25–35 10.1104/pp.121.1.25 10482657 PMC59374

